# Refractory secondary glaucoma-clinical case

**Published:** 2012-03-05

**Authors:** IR Barac, MD Pop, F Baltă

**Affiliations:** * Department of Ophthalmology, “Carol Davila” University of Medicine and Pharmacy, Bucharest

**Keywords:** glaucoma, oil silicon, Ahmed valve

## Abstract

**Rationale and objective:**The major objective of treatment in glaucomatous disease is the decrease of intraocular pressure while maintaining the patient's vision and quality of life. Despite therapeutic possibilities, some cases of glaucoma remain refractory to treatment with the maintenance of elevated intraocular pressure and further progression of the disease.

Artificial drainage systems, Ahmed valve, is a treatment alternative for refractory glaucoma when medical therapy, laser or conventional surgery have shown no results.

**Methods and results: **We present the case of a patient presenting with refractive to medical treatment secondary glaucoma, following cataract surgery and vitrectomy for retinal detachment.

**Discussions: **One of the complications of vitreoretinal surgery is secondary glaucoma. Some of the patients with this type of glaucoma are unresponsive to conventional medical therapy. In such situations, a DPS implantation is needed such as an Ahmed valve in our case.

There are situations in which classical surgery-trabeculectomy - has no theoretical chance of success (in cases of neovascular glaucoma, secondary glaucoma, and inflammatory glaucoma post vitreoretinal surgery), [5,6]. Even though ASD are only used for refractory glaucoma, in this type of glaucoma, ADS can be used successfully as first line surgery.

**Abbreviations** ADS = artificial drainage system; IOP = intraocular pressure; BCVA = best correct visual acuity; CA inhibitor = carbonic anhydrase inhibitor; OCT = optical coherence tomography

## Introduction

Increased ocular pressure (over 22 mmHg) determines, in time, irreversible damage to the optic nerve.

During vitreoretinal surgery, silicone oil is sometimes used for a long term tamponade. This silicone oil is removed after 3-4 months, as it can give rise to various complications, including glaucoma. The mechanism described in the development of glaucoma in patients with silicone oil tamponade consists of silicone oil pupillary block [**3,4**].

Even after these patients have had peripheral iridectomy, the development rate of secondary glaucoma is reported to be between 5.9% and 56%. [**1-3**] The mechanisms of increased intraocular pressure are complex, the blockage of the angle with emulsified oil being an important one. Most of these cases are handled by extracting the silicon oil (risk of retina redetachment) and management of glaucoma. Trabeculectomy has a poor prognosis in patients with removal of silicone oil because of the high risk of recurrence of retinal detachment due to hypotony.

## Case report

Male patient, age 64 years, presents in January 2011, with a diagnosis of refractory secondary glaucoma.

Ophthalmologic history:

- BE small myopia( RE -1.25DSf ; LE-3DSf)

- November 2009 RE Cataract surgery with PC IOL

- November 2009 RE (after cataract surgery) develops retinal detachment; retinal detachment is solved by pars plana vitrectomy and endotamponament with silicone oil. 

- July 2010 silicone oil is removed and intraocular hypertension is diagnosed

Between November and July the patient had no ocular pain. The medical treatment for glaucoma succeeded in compensating pressure values. The patient was admitted for investigation and conduct therapeutic setting.

Clinical examination at 8 A.M. in **January 2011:**

- BCVA RE 0,2; BCVA LE 0,8

- IOP RE 43 mmHg; LE 20 mmHg with topical treatment (beta-blocker, analogue of prostaglandin).

- Pahimetry RE AVG 575, corrected IOP -2; LE AVG 567, corrected IOP -1

- Visual field RE: narrowed nasal 10-15 degrees, superior 30 degrees, temporal 60 degrees, inferior 30 degrees; LE normal.

- OCT RNFL RE decreased in all 4 quadrants; LE deceased nasal and inferior (due to myopia)

- Ultrasound examination: RE attached retina, traces of silicone oil.

We added to the treatment carbonic anhydrate inhibitor (eye drops form) and obtained IOP RE 20 mmHg, corrected IOP 18 mmHg pressure measured at 12 hours.

After one month, in **February 2011:**

Clinical examination at 8 in the morning:

- BCVA RE 0,2; BCVA LE 0,8.

- IOP RE 34 mmHg; LE 20 mmHg with topical treatment (beta-blocker, analogue of prostaglandin, CA inhibitor, alpha-blocker).

- Pahimetry RE AVG 575, corrected IOP -2; LE AVG 567, corrected IOP -1

- Visual field RE narrowed nasal 10 degrees, superior 30 degrees, temporal 50 degrees, inferior 30 degrees; LE normal.

- Gonioscopy: of the RE revealed multiple anterior synechiae and pigmentary dispersion in nasal, inferior, temporal fields and emulsified silicon oil in the superior field. LE multiple anterior synechiae nasal and pigmentary dispersion in inferior, temporal and superior fields.

The next day, at 8 AM, RE IOP = 32 mmHg after receiving the same local treatment.

Fluctuations of the intraocular pressure were observed during the day with elevated IOP in the morning and the presence of emulsified oil at a slight angle examination. Patient also presented visual field loss [**7,8**].

We decided to implant an Ahmed valve in the RE. The usual technique with positioning the implant in the superior temporal quadrant with scleral flap (patient had a good conjunctiva and sclera) is used. 

Postoperative, no complications were noted. Patient presented dyplopia during eye movement that disappeared in the primary position of the eyeballs. 

At discharge, the valve was covered with conjunctiva; the tube was present in the anterior chamber, with clear aqueous humor. The valve was functional, RE IOP 10 mmHg without medication.

The recommendations were topical treatment with antibiotic and corticosteroid at RE; at LE, anti glaucoma medical treatment with prostaglandin analogues associated with beta-blockers and carbonic anhydrase inhibitors.

After one month, **March 2011:**

- BCVA RE 0,2 ; BCVA LE 0,8

- IOP RE 12 mmHg (without treatment) LE 18 mmHg with topical treatment (beta-blocker, analogue of prostaglandin, CA inhibitor)

- Visual field BE is the same

- Gonioscopy: RE multiple anterior synechiae and pigmentary dispersion in nasal, inferior and temporal fields; emulsified silicon oil in the superior field; LE multiple anterior synechiae nasal, pigmentary dispersion inferior, temporal and superior fields.

- Anterior pole RE: Ahmed valve covered with conjunctiva, functional filtering bulb, the tube present in anterior chamber, unblocked by emulsified silicon oil, transparent cornea, clear aqueous humor, without dyplopia.

- Ultrasound examination: RE attached retina

## Discussion

This is the case of a patient with low grade myopia, who, after cataract surgery, developed retinal detachment. The retina was successfully reattached but, as a complication of intraocular presence of silicone oil, secondary glaucoma developed. Glaucoma did not respond to traditional medication.

The IOP was elevated in the morning, probably related to the supine position and the presence of emulsified oil bubbles at the angle. The particularity of the case is that the patient had a cluster angle, with pigmentary dispersion and anterior synechiae at BE. An Ahmed valve implanted was successfully performed, using a traditional operative technique; postoperative complications were minimal and resolved.

## Conclusions

The Ahmed valve may be a solution for patients with secondary glaucoma after vitroretinal surgery when medication is not effective. Because of the valve mechanism, intraocular pressure does not decrease as fast as it does after trabeculectomy, so the risk of retinal redetachment is lower. Most surgeons recommend Ahmed valve as a last therapeutic solution for refractory glaucoma after repeated trabeculectomy. During the three decades that have passed after the approval of this device, developed by Molteno, the indications for artificial devices changed. Currently, artificial drainage systems can be used successfully for secondary glaucoma after vitreoretinal surgery, neovascular glaucoma and inflammatory glaucoma.

## References

[1] Ishida  K, Ahmed  II, Netland  PA (2009). Ahmed Glaucoma Valve surgical outcomes in eyes with and without silicone oil endotamponade. J Glaucoma.

[2] Ando  F (1985). Intraocular hypertension resulting from papillary block by silicone oil. Am J Ophthalmology.

[3] Jonas  JB, Knorr  HL, Rank  RM (2001). Intraocular pressure and silicon oil endotamponade. J Glaucoma.

[4] Al Jazzaf  AM, Netland  PA, Charles  S (2005). Incidence and management of elevated intraocular pressure after silicone oil injection. J Glaucoma.

[5] Shaarawy  MM, Sherwood  MB, Hitchings  RA (2009). Glaucoma surgical management.

[6] Yanoff  M, Duker  JS (2004). Ophthalmology, second edition.

[7] Caprioli , Coleman  (2008). Intraocular pressure fluctuation: A risk factor for visual field progression at low intraocular pressure in the advanced glaucoma intervention study. Ophthalmology.

[8] Weinreb  RN, Makoto  A, Remo  S Medical Treatment of Glaucoma.

